# CircUBAP2-mediated competing endogenous RNA network modulates tumorigenesis in pancreatic adenocarcinoma

**DOI:** 10.18632/aging.102334

**Published:** 2019-10-04

**Authors:** Rongjie Zhao, Junjie Ni, Si Lu, Sujing Jiang, Liangkun You, Hao Liu, Jiawei Shou, Chongya Zhai, Wei Zhang, Shengpeng Shao, Xinmei Yang, Hongming Pan, Weidong Han

**Affiliations:** 1 Department of Medical Oncology, Sir Run Run Shaw Hospital, College of Medicine, Zhejiang University, Hangzhou 310000, Zhejiang, China; 2 The First Clinical College, Zhejiang Chinese Medical University, Hangzhou 310053, Zhejiang, China; 3 The Fourth Clinical College, Zhejiang Chinese Medical University, Hangzhou 310053, Zhejiang, China; 4 Department of Radiation and Medical Oncology, The First Affiliated Hospital of Wenzhou Medical University, Wenzhou 325000, Zhejiang, China; 5 The Second Clinical College, Zhejiang Chinese Medical University, Hangzhou 310053, Zhejiang, China; 6 Department of Oncology, The First Affiliated Hospital of Jiaxing University, Jiaxing 314000, Zhejiang, China

**Keywords:** pancreatic adenocarcinoma, circRNA, ceRNA, immune microenvironment

## Abstract

We investigated the role of the competing endogenous RNA (ceRNA) network in the development and progression of pancreatic adenocarcinoma (PAAD). We analyzed the expression profiles of PAAD and normal pancreatic tissues from multiple GEO databases and identified 457 differentially expressed circular RNAs (DEcircRNAs), 19 microRNAs (DEmiRNAs) and 1993 mRNAs (DEmRNAs). We constructed a ceRNA network consisting of 4 DEcircRNAs, 3 DEmiRNAs and 149 DEmRNAs that regulates the NF-kappa B, PI3K-Akt, and Wnt signaling pathways. We then identified and validated five hub genes, *CXCR4*, *HIF1A*, *ZEB1*, *SDC1* and *TWIST1*, which are overexpressed in PAAD tissues. The expression of *CXCR4*, *HIF1A*, *ZEB1,* and *SDC1* in PAAD was regulated by *circ-UBAP2* and *hsa-miR-494*. The expression of *CXCR4* and *ZEB1* correlated with the levels of M2 macrophages, T-regulatory cells (Tregs) and exhausted T cells in the PAAD tissues. The expression of *CXCR4* and ZEB1 positively correlated with the expression of *CTLA-4* and *PD-1*. This suggests that *CXCR4* and *ZEB1* proteins inhibit antigen presentation and promote immune escape mechanisms in PAAD cells. In summary, our data suggest that the *circUBAP2*-mediated ceRNA network modulates PAAD by regulating the infiltration and function of immune cells.

## INTRODUCTION

Pancreatic adenocarcinoma (PAAD) is the most commonly occurring pancreatic cancer, and the seventh leading cause of tumor-related death in both men and women worldwide [1]. It is often asymptomatic during the early stages. Although the treatment of PAAD has improved in recent years, thanks to the availability of newer chemotherapeutic drugs and advances in surgical techniques, the survival rates of PAAD patients remain very low. The majority of the patients are diagnosed with advanced stage PAAD, which is not amenable for surgical therapy [2]. Moreover, the 5-year survival rates of patients that have undergone successful surgery remain below 8% because of disease recurrence [3]. In addition, high rates of drug resistance to several chemotherapeutic drugs, including gemcitabine also contribute to low survival rates [4]. Therefore, reliable biomarkers useful for accurate early diagnoses are needed to improve survival rates in patients with PAAD.

Previous studies have revealed several protein-coding genes, miRNAs or long non-coding RNAs (lncRNAs) that regulate PAAD. For example, upregulation of *miR-222* promotes tumor cell proliferation and invasiveness in PAAD by decreasing the nuclear levels of the *p27* tumor suppressor protein [5]. In addition, silencing *LINC00958* prevents initiation of pancreatic tumorigenesis by inhibiting *PAX8* via *miR-330-5p* [6]. Circular RNAs (circRNAs) are a novel class of endogenous small non-coding RNAs that form covalently closed-loop structures and lack a 5′ cap or 3′ poly-A tail [7]. Although previously thought to be generated as a result of splicing errors [8], recent advances in RNA sequencing technologies have revealed that they play a key role in regulating gene expression by sponging miRNAs [9]. Aberrant expression of circRNAs has been documented in several human diseases, including cancers [10].

The exosomes secreted by PAAD cells contain circular aminoacyl-tRNA synthetases (*circ-IARS)*, which promote tumor invasion and metastasis by enhancing endothelial monolayer permeability [11]. A competing endogenous RNA (ceRNA) network that includes circRNAs and miRNAs plays a critical role in posttranscriptional regulation of key proteins, and dysregulation of ceRNAs is associated with tumor development and progression [12]. However, the role of circRNAs in the growth and progression of PAAD is not fully understood [13]. The immune system plays a crucial role in PAAD growth and progression [14]. For that reason, we investigated the relationship between ceRNAs and tumor-infiltrated immune cells in the development and progression of PAAD.

## RESULTS

### Identification of DEcircRNAs, DEmiRNAs, and DEmRNAs in PAAD tissues

**Figure 1:**
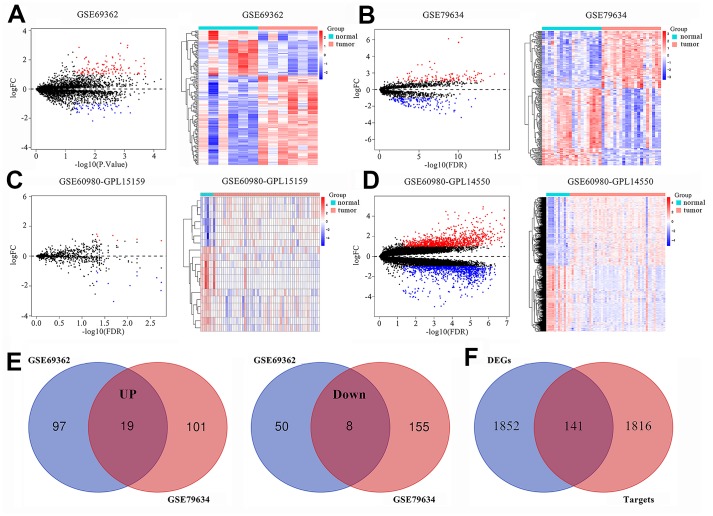
**Differentially expressed circRNAs, miRNAs and mRNAs in pancreatic adenocarcinomas (PAAD).** Volcano plots and heatmaps show identification of (**A**) DEcircRNAs in GSE69362, (**B**) DEcircRNAs in GSE79634, (**C**) DEmiRNAs in GSE60980 (GPL15159) and (**D**) DEmRNAs in GSE60980 (GPL14550) between PAAD tissues and adjacent normal pancreatic tissue. The red color indicates upregulated genes in the PAAD tissues and blue color indicates downregulated genes, while black color indicates genes with no significant differences between the PAAD and normal pancreatic tissues. Heatmaps show the expression patterns of DEcircRNAs, DEmiRNAs and DEmRNAs. The PAAD and adjacent normal pancreatic tissues are represented by red and blue color, respectively. (**E**) Venn diagrams show commonly upregulated DEcircRNAs and downregulated DEcircRNAs in the PAAD tissues in both GSE69362 and GSE79634. Purple and orange circles indicate the number of DEcircRNAs in the GSE69362 and GSE79634 datasets, respectively. The red circles in the middle indicate the overlapping circRNAs between the two datasets. (**F**) Venn diagram shows the intersection between DEmiRNA-predicted targets obtained from miRDB, miRTarBase and TargetScan databases and DEmRNAs in GSE60980 (GPL14550).

We normalized the expression of circRNAs (Supplementary Figures 1A and 1B), microRNAs and mRNAs (Supplementary Figures 1C and 1D) in PAAD and normal pancreatic tissues. We identified 457 differentially expressed circRNAs (DEcircRNAs), which included 174 in the GSE69362 dataset [15] and 283 in the GSE79634 dataset [16], based on a threshold of *P* value < 0.05 and |log FC| ≥ 1. We generated volcano plots and heatmaps of the DEcircRNAs (Figures 1A and 1B), 19 DEmiRNAs (7 up-regulated and 12 down-regulated; Figure 1C) and 1993 DEmRNAs (1012 up-regulated and 981 down-regulated; Figure 1D) to demonstrate their aberrant expression in PAAD tissues. We compared the DEcircRNAs from the two datasets and identified 19 up-regulated and 8 down-regulated DEcircRNAs for further analysis (Figure 1E).

### Construction of the circRNA-miRNA-mRNA network

**Figure 2:**
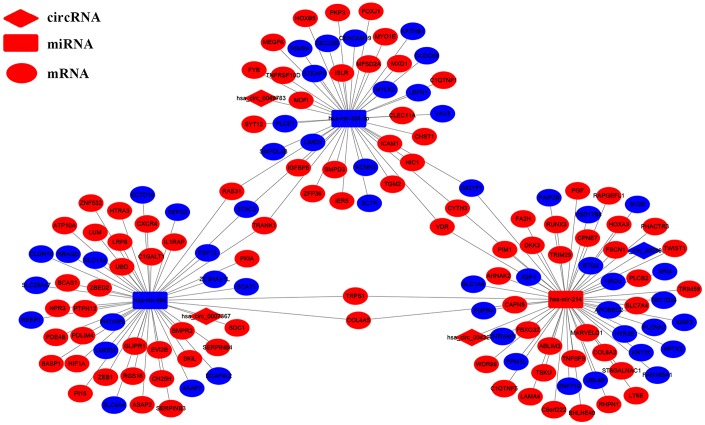
**The circRNA-miRNA-mRNA interaction network in the PAAD tissues.** The circRNA-miRNA-mRNA interaction network consists of 4 circRNAs (*hsa_circ_0007367*, *hsa_circ_0003958*, *hsa_circ_0043278* and *hsa-circ-0049783*), 3 miRNAs (*hsa-mir-324-3p*, *hsa-miR-214* and *hsa-miR-494*), and 149 mRNAs. The diamond nodes indicate the circRNAs; rectangle nodes indicate the miRNAs; the elliptical nodes indicate the mRNAs. The edges indicate a possible connection between the circRNAs, miRNAs, and the mRNAs. The red and blue color indicates high and low expression of the ceRNAs in the PAAD tissues, respectively.

**Figure 3:**
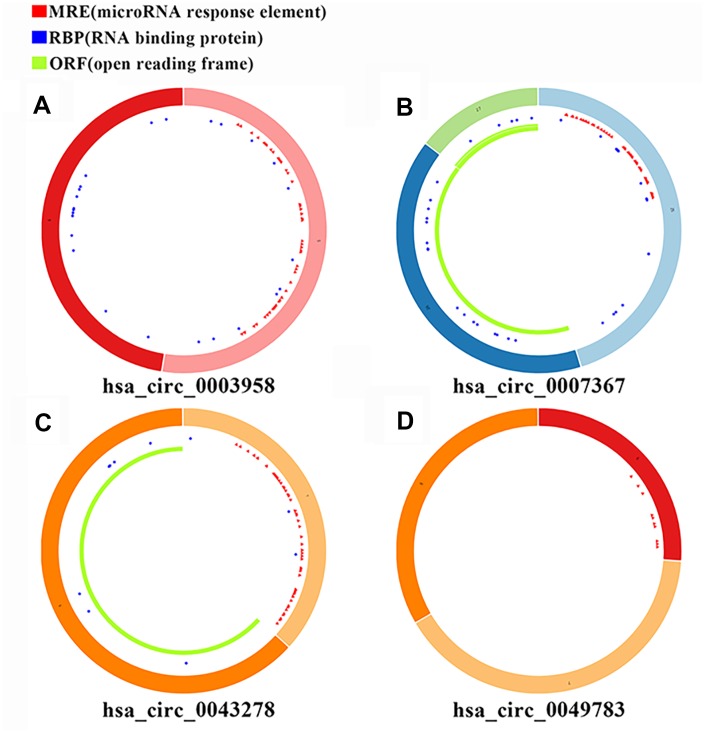
**Basic structural features of the 4 PAAD-specific circRNAs.** The structural features of (**A**) *hsa_circRNA_0003958*, (**B**) *hsa_circRNA_0007367*, (**C**) *hsa_circRNA_0043278*, and (**D**) *hsa_circRNA_0049783* downloaded from the Cancer-Specific CircRNA Database (CSCD) are shown. The microRNA response element (MRE) is shown in red; The RNA binding protein (RBP) is shown in blue; The open reading frame (ORF) is shown in green.

We analyzed the DEcircRNAs, DEmiRNAs and DEmRNAs using the CSCD database and identified circRNA-miRNA pairs. We also identified miRNA-mRNA pairs by analyzing the miRDB, miRTarBase and TargetScan databases (Figure 1F). Finally, we generated a ceRNA network that consisted of 4 DEcircRNAs (*circ-HIBADH*, *circ-UBAP2*, *circ-TADA2A* and *circ-CLEC17A*), 3 DEmiRNAs (*has-miR-214*, *has-mir-324-3p* *and* *has-miR-494*) and 149 DEmRNAs using the Cytoscape software as shown in Figure 2. The basic characteristics of the 4 DEcircRNAs are shown in Table 1 and Figure 3. Furthermore, we found that *circ-CLEC17A* is aberrantly overexpressed in PAAD tissues from females compared to males (Supplementary Figures 2A–2D).

**Table 1:** Basic characteristics of the 4 differently expressed circRNAs.

**CircRNA**	**Log2FC**	**Regulation**	**Type**	**Chromosome**	**Strand**	**Gene symbol**
hsa_circ_0049783	1.35	UP	exonic	chr19	+	*CLEC17A*
hsa_circ_0007367	1.34	UP	exonic	chr9	-	*UBAP2*
hsa_circ_0003958	1.01	DOWN	exonic	chr7	-	*HIBADH*
hsa_circ_0043278	1.19	UP	exonic	chr17	+	*TADA2A*

Log2FC means that log2 transformation to fold changes of circRNA expression between PAAD tissues and normal tissues. UP means upregulated circRNA in PAAD tissues. DOWN means downregulated circRNA in PAAD tissues.

### Analysis of DEmRNAs

**Figure 4:**
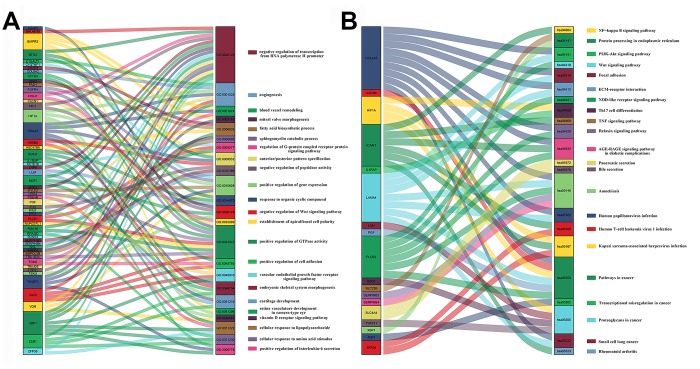
**Sankey plots of signaling pathways and biological processes related to differentially expressed mRNAs in PAAD tissues.** Sankey plots show (**A**) signaling pathways and (**B**) biological processes (BP) related to DEmRNAs involved in PAAD based on Kyoto Encyclopedia of Genes and Genomes (KEGG) enrichment and Gene Ontology_Biological Process (GO_BP) analyses, respectively.

We performed Gene ontology (GO) and Kyoto Encyclopedia of Genes and Genomes (KEGG) analyses of the DEmRNAs to identify aberrantly regulated biological processes and signaling pathways in PAAD. The Gene Ontology analyses suggested that biological processes such as cell proliferation, angiogenesis and negative regulation of transcription from RNA polymerase II promoter were dysregulated (Figure 4A). KEGG pathway analysis of DEmRNAs highlighted the involvement of the NF-kappa B, PI3K-Akt, and Wnt signaling pathways, and cancer-related dysregulation of transcription (Figure 4B). The NF-kappa B, PI3K-Akt, and Wnt signaling pathways have previously been implicated in the growth and progression of PAAD [17–19]. The Sankey plots for all the enriched GO terms and KEGG pathways with a *P* value < 0.05 were shown, further highlighting the genes and the pathways involved in PAAD.

### Identification and validation of the hub genes

**Figure 5:**
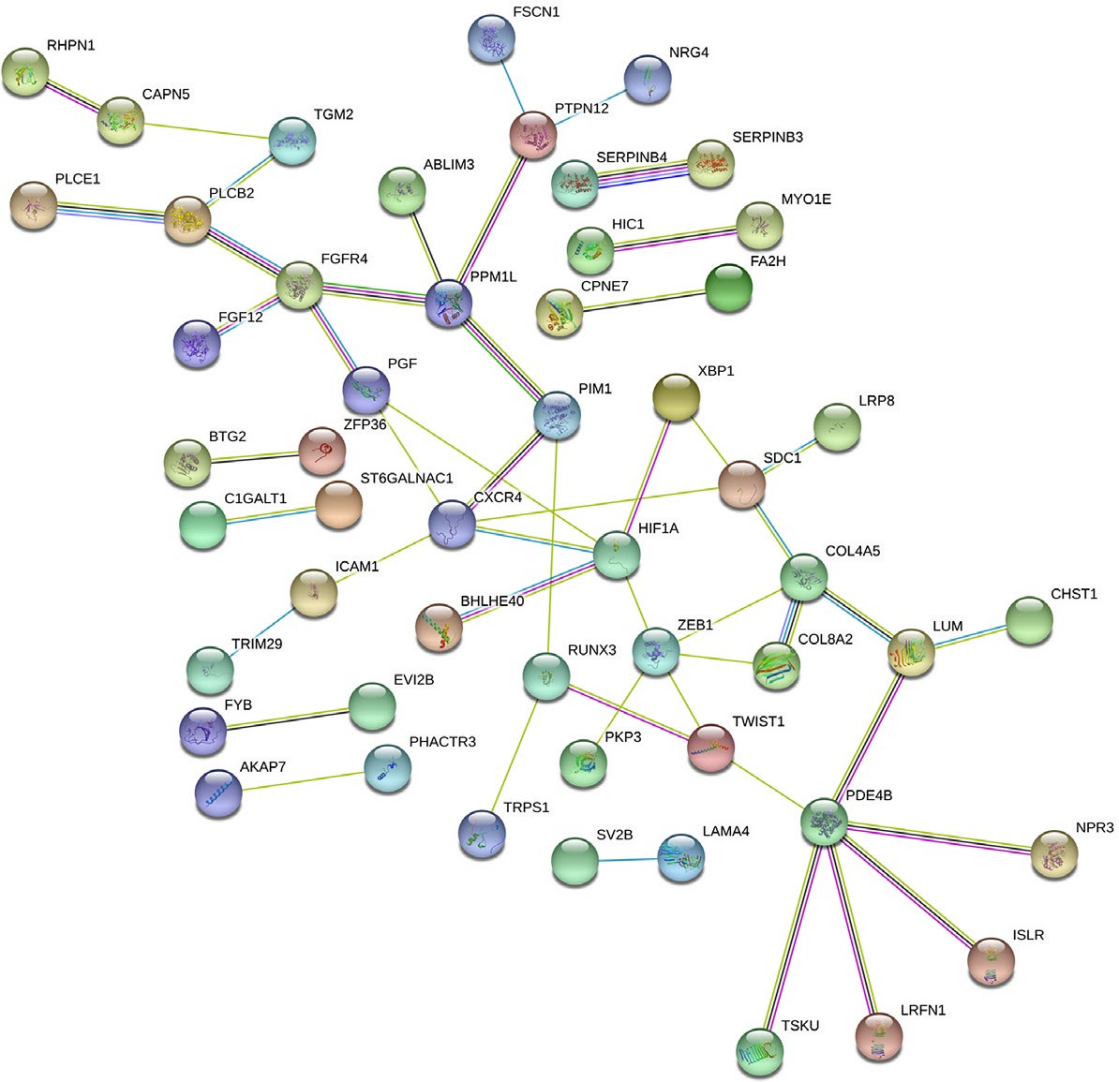
**Protein-protein intersection (PPI) network analyses of the DEmRNAs involved in the ceRNA network.** The PPI network consists of 141 edges and 50 nodes representing proteins and interactions, respectively. The relative thickness of the edges represents the degree of relationship (weak, moderate, or strong) between the nodes.

We analyzed the DEmRNAs using the Search Tool for the Retrieval of Interacting Genes database (STRING) and constructed a protein-protein interaction (PPI) network consisting of 141 nodes and 50 edges (Figure 5). We identified the top 15 genes, which were found in at least 6/11 topological algorithms and had a high-ranking score. After integrating the results of the GO, KEGG and PPI network analyses, we identified 5 hub genes that were closely related to PAAD tumorigenesis, namely, C-X-C Motif Chemokine Receptor 4 (*CXCR4*), Hypoxia-Inducible Factor 1 Subunit Alpha (*HIF1A*), Zinc Finger E-Box Binding Homeobox 1 (*ZEB1*), Syndecan 1 (*SDC1*) and Twist Family BHLH Transcription Factor 1 (*TWIST1*).

**Figure 6:**
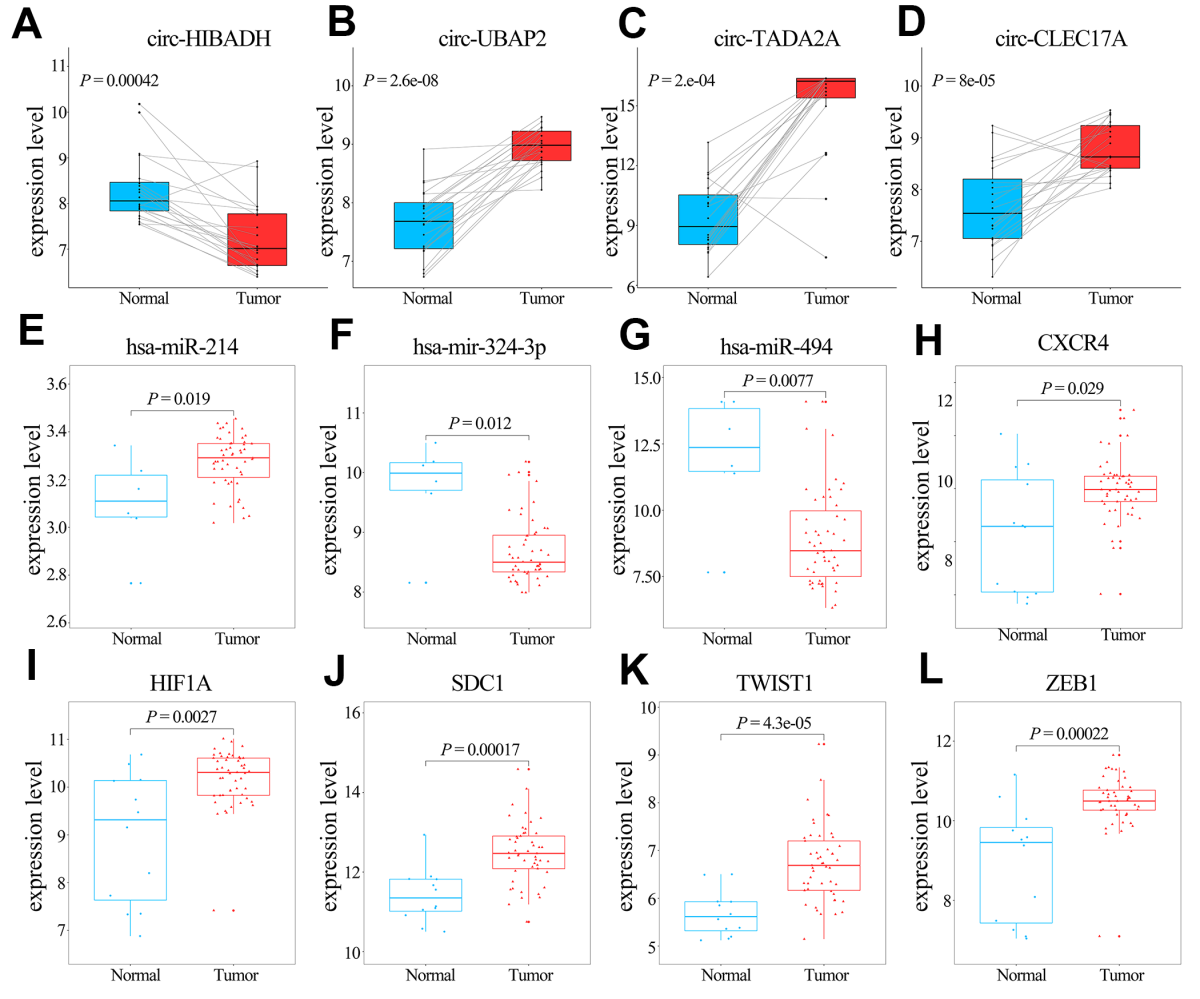
**Comparative analyses of the transcription levels of hub genes in GSE60980 (GPL14550), and related miRNAs in GSE60980 (GPL15159) and circRNAs in GSE79634 between PAAD and normal pancreatic tissues.** (**A**–**K**) Transcription levels of *circ-HIBADH*, *circ-UBAP2*, *circ-TADA2A*, *circ-CLEC17A*, *hsa-miR-214*, *hsa-mir-324-3p*, *hsa-miR-494*, *CXCR4*, *HIF1A*, *SDC1*, *TWIST1* and *ZEB1* between PAAD (red) and normal pancreatic tissues (blue).

**Figure 7:**
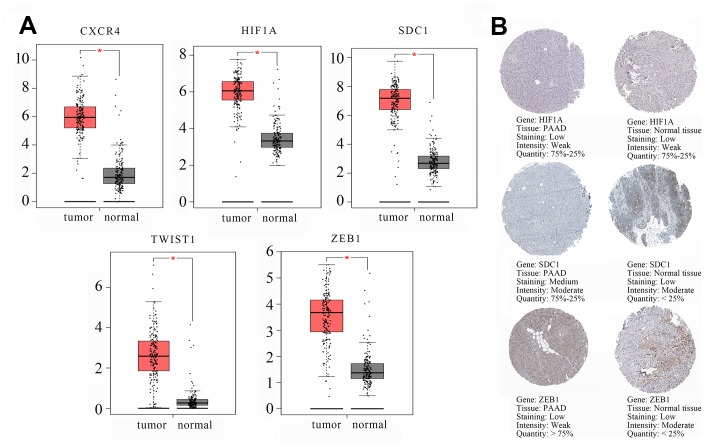
**Validation of the transcript (mRNA) and protein levels of the hub genes using the Gene Expression Profiling Interactive Analysis (GEPIA) and The Human Protein Atlas database.** (**A**) The transcript (mRNA) of the hub genes, namely, *CXCR4*, *HIF1A*, *SDC1*, *TWIST1*, and *ZEB1* in PAAD and normal pancreatic tissues are shown. (**B**) Immunohistochemical (IHC) staining data of hub genes as follows: *HIF1A* protein expression in a PAAD tumor tissue (Staining: low; Intensity: weak; Quantity: 75%-25%; Location: cytoplasmic/membrane); *HIF1A* protein expression in a representative normal pancreatic tissue (Staining: low; Intensity: weak; Quantity: 75%-25%; Location: nuclear); *SDC1* protein expression in a PAAD tumor tissue (Staining: medium; Intensity: moderate; Quantity: 75%-25%; Location: cytoplasmic/membrane). *SDC1* protein expression in a representative normal pancreatic tissue (Staining: low; Intensity: moderate; Quantity: < 25%; Location: cytoplasmic/membrane). *ZEB1* protein expression in a PAAD tumor tissue (Staining: low; Intensity: weak; Quantity: > 75%; Location: cytoplasmic/membrane). *ZEB1* protein expression in a representative normal pancreatic tissue (Staining: low; Intensity: moderate; Quantity: < 25%; Location: cytoplasmic/membrane). The database lacked information regarding *CXCR4* and *TWIST1* protein expression in PAAD tumor tissues and normal pancreatic tissues. IHC results consistent with changed trend of transcript (mRNA) of hub genes in GSE60980 (GPL14550) and GEPIA were displayed.

The transcription levels of the 5 hub genes in GSE60980 (GPL14550), 3 related miRNAs in GSE60980 (GPL15159), and 4 circRNAs in GSE79634 (Figure 6) and GSE69362 (Supplementary Figure 3) were shown. We further validated the 5 hub genes by analyzing their mRNA expression in the Gene Expression Profiling Interactive Analysis (GEPIA) and the protein expression in The Human Protein Atlas (THPA) database as shown in Figures 7A and 7B, respectively. The transcription levels of these 5 hub genes in GEPIA were similar to the results in Figure 6. The protein expression data in the THPA database showed that *SDC1* and *ZEB1* were aberrantly overexpressed in PAAD compared to normal pancreatic tissues, which were in accordance with our transcription results in Figure 6. However, *HIF1A* protein levels were low in both PAAD and normal pancreatic tissues, and the protein levels of the remaining two hub proteins, *CXCR4* and *TWIST1* were not found in the THPA database.

### Analysis of prognosis and tumor infiltration of immune cells

**Figure 8:**
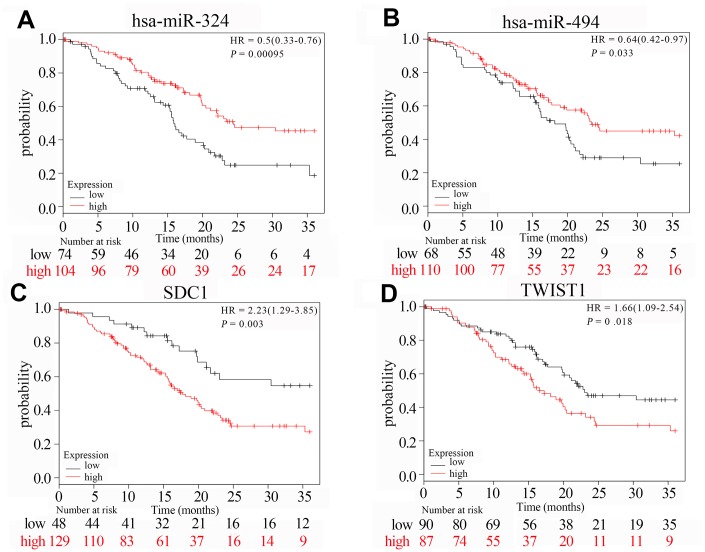
**The survival curves of hub genes and their related miRNAs based on Kaplan Meier.** (**A**–**D**) Transcription levels of *hsa-miR-324*, *hsa-miR-494*, *SDC1* and *TWIST1* are significantly related to the overall survival of patients with PAAD (*P* < 0.05). Red curve indicates high expression in PAAD tumor tissues; black curve indicates low expression in PAAD tumor tissues; *P* value < 0.05 indicates statistical significance; HR: hazard ratio.

We analyzed the overall survival (OS) using the Kaplan-Meier Plotter database (KM plotter database) to determine the prognostic value of hub genes and miRNAs. Prognosis based on their high and low expression in PAAD tissues is shown in Figure 8. High expression of *SDC1* and *TWIST1* was associated with poorer OS (HR = 2.23, *P* = 0.0030 and HR = 1.66, *P* = 0.018). High expression of *hsa-miR-494* and *hsa-miR-324* was associated with increased OS (HR = 0.64, *P* = 0.033 and HR = 0.50, *P* = 0.00095).

Since several studies have shown that immune cell infiltration and the tumor microenvironment plays a critical role in the development of cancers, we analyzed the relationship between the expression of the hub genes and the status of immune cell infiltration in PAAD tumors using the Tumor Immune Estimation Resource (TIMER) database. As shown in Supplementary Figure 4, *CXCR4* expression positively correlated with the infiltration levels of B cells (Cor = 0.401, *P* = 5.40e-08), CD4+ T cells (Cor = 0.435, *P* = 3.56e-09), neutrophils (Cor = 0.496, *P* = 5.46e-12), macrophages (Cor = 0.467, *P* = 1.20e-10), and dendritic cells or DCs (Cor = 0.503, *P* = 2.40e-12). *ZEB1* expression positively correlated with infiltration levels of CD8+ T cells (Cor = 0.525, *P* = 1.59e-13), neutrophils (Cor = 0.519, *P* = 3.44e-13), macrophages (Cor = 0.693, *P* = 8.68e-26), and DCs (Cor = 0.572, *P* = 3.09e-16). However, *SDC1* expression showed no significant correlation with immune cell infiltration in the PAAD tumors. These results suggested that the circRNA-mediated ceRNAs might modulate tumorigenesis by regulating the infiltration of immune cells in PAAD.

### Correlation analysis between the hub genes and the immune cell markers

Next, to further characterize the role of the different subsets of immune cells with PAAD tumorigenesis, we analyzed the correlation between the expression of hub genes and various immune cell markers, including markers specific for CD8+ T cells, T cells (general), B cells, monocytes, tumor-associated macrophages (TAMs), M1 and M2 macrophages, neutrophils, natural killer cells (NKs) and DCs. We also analyzed the data for different subsets of T cells, such as T-helper 1 (Th1) cells, T-helper 2 (Th2) cells, follicular helper T cells (Tfhs), T-helper 17 cells (Th17s), regulatory T cells (Tregs) and exhausted T cells. After adjusting for purity, we found that the expression of most of the immune cell markers positively correlated with the expression of *CXCR4* and *ZEB1* and negatively correlated with *SDC1* expression in PAAD tissues (Table 2).

**Table 2:** Correlation analyses between 5 hub genes and the corresponding markers of immune cells in TIMER.

**Description**	**Gene makers**	**CXCR4**		**HIF1A**		**SDC1**		**TWIST1**		**ZEB1**	
		**Purity**		**Purity**		**Purity**		**Purity**		**Purity**	
		**Cor**	***P***	**Cor**	***P***	**Cor**	***P***	**Cor**	***P***	**Cor**	***P***
CD8+ T cell	CD8A	0.667	***	0.265	***	-0.423	***	-0.025	0.749	0.259	***
	CD8B	0.549	***	0.188	*	-0.273	***	-0.039	0.609	0.419	***
T cell (general)	CD3D	0.727	***	0.119	0.120	-0.304	***	0.080	0.299	0.367	***
	CD3E	0.739	***	0.181	*	-0.344	***	0.051	0.510	0.447	***
	CD2	0.711	***	0.207	**	-0.377	***	0.028	0.718	0.465	***
B cell	CD19	0.612	***	0.020	0.800	-0.135	0.077	0.126	0.100	0.352	***
	CD79A	0.610	***	0.102	0.184	-0.147	0.056	0.122	0.112	0.344	***
Monocyte	CD86	0.665	***	0.425	***	-0.273	***	0.321	***	0.552	***
	CD115 (CSF1R)	0.592	***	0.360	***	-0.415	***	0.107	0.163	0.598	***
TAM	CCL2	0.394	***	0.320	***	-0.384	***	0.037	0.629	0.335	***
	CD68	0.386	***	0.319	***	-0.019	0.805	0.280	***	0.253	***
	IL10	0.527	***	0.297	***	-0.230	**	0.250	***	0.501	***
M1 macrophage	INOS (NOS2)	0.193	*	0.128	0.095	-0.091	0.237	0.177	*	0.191	*
	IRF5	0.307	***	-0.152	*	0.081	0.291	0.201	**	0.047	0.540
	COX2(PTGS2)	0.030	0.699	0.292	***	0.226	**	0.281	***	0.224	**
M2 macrophage	CD163	0.541	***	0.424	***	-0.351	***	0.179	*	0.594	***
	VSIG4	0.484	***	0.321	***	-0.349	***	0.213	**	0.472	***
	MS4A4A	0.602	***	0.355	***	-0.393	***	0.202	**	0.579	***
Neutrophils	CD66b (CEACAM8)	0.189	*	0.079	0.303	-0.085	0.269	0.022	0.772	0.099	0.196
	CD11b (ITGAM)	0.490	***	0.238	**	-0.208	**	0.261	***	0.409	***
	CCR7	0.701	***	0.151	*	-0.314	***	0.035	0.647	0.424	***
Natural killer cell	KIR2DL1	0.235	**	0.103	0.180	-0.058	0.450	0.118	0.123	0.147	0.055
	KIR2DL3	0.279	***	0.108	0.158	-0.043	0.577	0.111	0.149	0.185	*
	KIR2DL4	0.056	0.469	0.099	0.197	-0.081	0.290	0.056	0.469	0.001	0.990
	KIR3DL1	0.305	***	0.168	*	-0.253	***	0.029	0.708	0.226	**
	KIR3DL2	0.239	**	0.032	0.679	-0.120	0.119	0.184	*	0.181	*
	KIR3DL3	-0.074	0.334	0.056	0.464	0.043	0.577	0.052	0.500	0.115	0.135
	KIR2DS4	0.073	0.340	0.070	0.360	-0.116	0.132	-0.026	0.733	0.030	0.702
Dendritic cell	HLA-DPB1	0.721	***	0.244	**	-0.306	***	0.146	0.056	0.466	***
	HLA-DQB1	0.457	***	0.171	*	-0.245	**	0.092	0.231	0.295	***
	HLA-DRA	0.685	***	0.341	***	-0.298	***	0.209	**	0.513	***
	HLA-DPA1	0.671	***	0.303	***	-0.320	***	0.147	0.055	0.513	***
	BDCA-1(CD1C)	0.618	***	0.220	**	-0.304	***	0.002	0.987	0.460	***
	BDCA-4(NRP1)	0.376	***	0.645	***	-0.393	***	0.041	0.594	0.660	***
	CD11c (ITGAX)	0.617	***	0.266	**	-0.151	*	0.388	***	0.379	***
Th1	T-bet (TBX21)	0.662	***	0.174	*	-0.386	***	-0.017	0.829	0.372	***
	STAT4	0.602	***	0.229	**	-0.492	***	-0.116	0.132	0.413	***
	STAT1	0.164	*	0.369	***	0.004	0.961	0.115	0.135	0.299	***
	IFN-γ (IFNG)	0.360	***	0.193	*	-0.096	0.213	0.127	0.098	0.223	**
	TNF-α (TNF)	0.409	***	0.106	0.168	-0.155	*	0.265	***	0.168	*
Th2	GATA3	0.175	*	0.064	0.407	0.180	*	0.224	**	0.098	0.204
	STAT6	-0.039	0.610	-0.013	0.869	0.092	0.232	-0.133	0.084	-0.085	0.271
	STAT5A	0.573	***	-0.064	0.402	-0.134	0.079	0.036	0.638	0.243	**
	IL13	0.144	0.060	-0.008	0.915	-0.123	0.109	0.012	0.888	0.144	0.060
Tfh	BCL6	0.264	***	0.447	***	-0.001	0.992	0.223	**	0.260	***
	IL21	0.343	***	0.152	*	-0.176	*	0.082	0.284	0.224	**
Th17	STAT3	0.341	***	0.526	***	-0.305	***	-0.003	0.966	0.563	***
	IL17A	0.108	0.161	0.202	**	-0.259	***	-0.184	*	0.119	0.122
Treg	FOXP3	0.612	***	0.227	**	-0.218	**	0.249	**	0.483	***
	CCR8	0.545	***	0.391	***	-0.242	**	0.182	*	0.585	***
	STAT5B	0.450	***	0.209	**	-0.469	***	-0.042	0.586	0.580	***
	TGFβ (TGFB1)	0.260	***	0.057	0.458	0.213	**	0.504	***	0.135	0.078
T cell exhaustion	PD-1 (PDCD1)	0.631	***	0.207	**	-0.225	**	0.085	0.269	0.327	***
	CTLA4	0.728	***	0.209	**	-0.276	***	0.194	*	0.394	***
	LAG3	0.426	***	0.033	0.673	-0.237	**	0.120	0.118	0.170	*
	TIM-3 (HAVCR2)	0.654	***	0.316	***	-0.239	**	0.330	***	0.492	***
	GZMB	0.484	***	0.214	**	-0.260	***	0.121	0.114	0.277	***

The following categories were used to define correlation strength based on the absolute values : “0.00–0.19” indicates very weak; “0.20–0.39” indicates weak; “0.40–0.59” indicates moderate; “0.60–0.79” indicates strong; “0.80–1.0” indicates very strong; *P* value < 0.05 was defined as statistically significant; Tfh, Follicular helper T cell; Tregs, regulatory T cells; Th, T helper cells; TAM, tumor-associated macrophages; Purity, correlation adjusted by purity; Cor, R-value of Spearman’s correlation; * *P* < 0.05; ** *P* < 0.01; *** *P* < 0.001.

**Figure 9:**
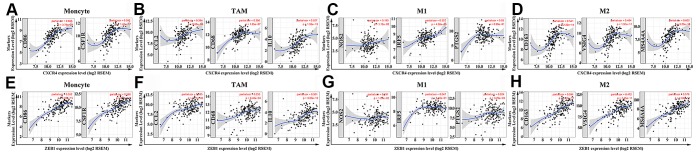
**The two hub genes positively correlate with macrophage polarization in PAAD tissues.** (**A**–**D**) The relationship between *CXCR4* expression and biomarkers of monocytes, TAMs, M1 and M2 macrophages is shown. (**E**–**H**) The relationship between *ZEB1* expression and biomarkers of monocytes, TAMs, M1 and M2 macrophages is shown. Note: Monocyte markers: *CD86* and *CSF1R*; TAM markers: *CCL2*, *CD68* and IL10; M1 macrophage markers: *NOS2*, *IRF5* and *PTGS2*; M2 macrophage markers: *CD163*, *VSIG4* and *MS4A4A*. Hub genes are shown on the x-axis, and the corresponding marker genes are shown on the y-axis.

The expression of specific markers for TAMs, M2 macrophages, monocytes and Tregs show a moderate to strong positive correlation with the expression of *CXCR4* and *ZEB1* (Table 2). These include TAM markers such as *CCL-2*, *CD68* and *IL10*, and M2 phenotype markers such as *CD163*, *VSIG4* and *MS4A4A* (Figure 9A–9H). Furthermore, the expression of specific markers for CD8+ T cells, T cells (general) and B cells show a strong positive correlation with the expression of *CXCR4* (Table 2). The high expression of DC markers, *HLA-DPB1*, *HLA-DQB1*, *HLA-DRA*, *HLA-DPA1*, *BDCA-1*, *BDCA-4* and *CD11c* correlates with increased expression of *CXCR4* (*P* <0.001) and *ZEB1* (*P* <0.001) (Table 2). This suggests that PAAD cells expressing high levels of *CXCR4* and *ZEB1* proteins might promote DC infiltration. We also observed positive correlation between *CXCR4* and markers of T cell exhaustion, such as programmed cell death protein 1 (*PD-1*), cytotoxic T lymphocyte-associated antigen-4 (*CTLA4*), lymphocyte-activation gene 3 (*LAG3*), T cell immunoglobulin domain and mucin domain-3 (*TIM-3*), and Granzyme B (*GZMB*) (*P* <0.001) (Table 2).

## DISCUSSION

Complex mechanisms involving several genes and pathways are involved in the growth and progression of PAAD. The ceRNA network has emerged a novel method of posttranscriptional gene regulation. In the present study, we identified a total of 457 DEcircRNAs in GSE69362 and GSE79634, 19 DEmiRNAs in GSE60980 (GPL15159) and 1993 DEmRNAs in GSE60980 (GPL14550). We generated a circRNA-mediated ceRNA network that included 4 circRNAs, 3 miRNAs and 149 mRNAs. We then constructed a PPI network and identified 5 hub genes, including *CXCR4*, *HIF1A*, *ZEB1*, *SDC1* and *TWIST1*, by performing functional enrichment using GO and KEGG pathway analyses. We further analyzed the relationship between the protein expression of the hub genes and the prognosis and the status of immune cell infiltration in PAAD.

We identified 4 circRNAs, *circ-UBAP2*, *circ-CLEC17A*, *circ-HIBADH* and *circ-TADA2A*, as part of the ceRNA network from among 457 DEcircRNAs. Previous studies have shown that *circ-UBAP2* plays an important role in many cancers by acting as a sponge for miRNAs. In triple-negative breast cancer (TNBC), upregulated *circ-UBAP2* binds to and inhibits *miR-661*, which promotes high expression of the *MTA1* oncogene that activates TNBC cell proliferation and migration [20]. In osteosarcoma cells, *circ-UBAP2* acts as an *miR-143* sponge and suppresses apoptosis by upregulating *Bcl-2* [21]. The role of *circ-UBAP2* in PAAD has not been reported. Our study suggests that up-regulated *circ-UBAP2* inhibits the expression of *hsa-miR-494* to promote pancreatic tumorigenesis. The roles of *circ-CLEC17A*, *circ-HIBADH* and *circ-TADA2A* in tumorigenesis have not been previously reported. Our results indicate that the *circ-CLEC17A*/*hsa-mir-324-3p*, *circ-HIBADH*/*hsa-miR-214* and *circ-TADA2A*/*hsa-miR-214* axis modulate the biogenesis, growth and progression of PAAD. Nevertheless, our data require further experimental confirmation.

We identified 5 hub genes that have previously been associated with PAAD tumorigenesis. Artemin promotes metastasis and invasiveness of PAAD cells by inducing *CXCR4* expression via the activation of the NF-κB signaling [22]. Moreover, *miR-494* inhibits proliferation, invasion and metastasis of prostate and breast cancer cells by suppressing *CXCR4* expression [23, 24]. Furthermore, *miR-494* suppresses epithelial-mesenchymal transition (EMT), metastasis and invasiveness of PAAD cells by downregulating *SDC1* [25]. Liu H et al. reported that downregulation of *TWIST1* suppresses PAAD cell invasiveness and metastasis [26]. *TWIST1* may be a direct target of *hsa-miR-214*, which is part of the ceRNA network we constructed in this study. Cao et al. reported that down-regulation of *mir-214-5p* promotes the proliferation, invasion and migration of PAAD cells in a *JAG1*-dependent manner, which is consistent with the effect of *TWIST1* knockdown [27]. We therefore speculate that the *hsa-miR-214*/*TWIST1* axis plays a critical role in PAAD progression. When exposed to hypoxic conditions, PAAD cells generate exosomes that are rich in *mir-301a-3p* which induces M2 polarization of the tumor-associated macrophages (TAMs) via the activation of PTEN/PI3Kγ in a *HIF1A*- or *HIF2A*-dependent manner, thereby promoting tumor cell EMT, invasiveness, and metastasis [28]. In hypoxic conditions, activated *KRAS* upregulates carbonic anhydrase 9 modulates pH and glycolysis via *HIF1A* or *HIF2A* and promotes aggressive growth of PAAD cells [29]. Notably, 4 out of the 5 hub genes, namely, *CXCR4*, *SDC1*, *ZEB1* and *HIF1A*, are potential targets of the *circ-UBAP2*/*hsa-miR-494* ceRNA network. This suggests that *circ-UBAP2*/*hsa-miR-494* ceRNA network plays a critical role in the initiation, growth, and progression of PAAD.

To address the potential regulatory roles of the hub genes in the recruitment of tumor-infiltrating lymphocytes, we assessed the correlation between the expression of hub genes and the status of immune infiltration levels in PAAD using the TIMER database. Our data suggest that high expression of both *CXCR4* and *ZEB1* correlates moderately or strongly with the markers of immune cell types such as M2 macrophages, *CD163*, *VSIG4* and *MS4A4A*, but shows a weak correlation with M1 macrophage markers. A previous study confirmed that high expression of *ZEB1* induces polarization of TAMs and promotes ovarian tumor growth [30]. We hypothesize that up-regulated *CXCR4* and *ZEB1* promotes M2 polarization of TAMs and promotes growth and progression of PAAD. Earlier studies have shown that activated Tregs suppress antitumor immunity and promote tumor survival [31]. In this study, Treg markers such as *FOXP3*, *CCR8* and *STAT5B* show a positive correlation with the mRNA levels of *CXCR4* and *ZEB1*. This suggests that *CXCR4* and *ZEB1* expression levels may indicate Treg activity in the tumor microenvironment. Santagata S et al. reported that the tumor suppressive activity of Tregs could be reversed by *CXCR4* inhibition in renal cancer cells [32]. Our results suggest that the high expression level of these two hub genes may contribute to recruitment and activation of Tregs in the PAAD microenvironment.

High expression of *CXCR4* and *ZEB1* correlates positively with critical immune checkpoint proteins such as *PD-1*, *CTLA4* and *TIM-3*. This suggests that high expression of *CXCR4* and *ZEB1* may induce T cell exhaustion and promote immune escape. Consistent with this hypothesis, Seo et al. reported that dual blockade of the *PD-1* and *CXCR4* pathways facilitates PAAD cell apoptosis via CD8+ T cells [33]. Garg et al. reported that NF-κB activity in pancreatic stellate cells induces high expression of *CXCL12*, a ligand of *CXCR4*, and this prevents the tumor infiltration of cytotoxic T cells and impairs their ability to kill tumor cells [34]. Furthermore, *CXCR4* inhibitor in combination with an immune-activating fusion protein called VIC-008 suppresses mesothelioma growth by inhibiting *PD-1* expression in CD8+ T cells and promotes transition of Tregs into T helper cells [35]. The upregulation of *PD-1* and *TIM-3* on CD4+ and CD8+ T cells restricts T cell responses in patients with PAAD [36].

In summary, our study demonstrates that circRNAs regulate PAAD by modulating the expression of the hub genes via the ceRNA network. Furthermore, the ceRNA network involving *circ-UBAP2*, *hsa-miR-494* and the 5 hub genes, especially for *CXCR4* and *ZEB1*, regulates PAAD by modulating the tumor infiltration of immune cells.

## MATERIALS AND METHODS

### Microarray data

In this study, the microarray data of circRNA was obtained from the GSE79634 and GSE69362 datasets. The GSE79634 dataset was based on the GPL19978 platform and included 20 PAAD and adjacent normal tissue samples each. The GSE69362 dataset was based on the GPL19978 platform and contained 6 PAAD and adjacent normal tissue samples each. The microarray data of miRNA was obtained from the GSE60980 (GPL15159) dataset and included 51 PAAD and 6 normal samples. The microarray data of mRNA was obtained from the GSE60980 (GPL14550) dataset and included 49 PAAD and 12 normal samples.

### Identification of DEcircRNAs, DEmiRNAs and DEmRNAs

The expression of cicrRNAs in the GSE79634 and GSE69362 datasets was normalized in quantile method, and the DEcircRNAs were determined using the "limma" package [37]. *P* values < 0.05 and |log FC| ≥ 1 were defined as statistically significant. Furthermore, to enhance the accuracy of the results, the DEcircRNAs were analyzed using the Venn diagram software (http://bioinformatics.psb.ugent.be/webtools/Venn/), and the intersections between the various DEcircRNAs were calculated. We used a similar strategy to analyze the differential expression of the miRNAs in GSE60980 (GPL15159) and the mRNAs in GSE60980 (GPL14550).

### Construction of the circRNA-miRNA-mRNA network

To construct the circRNA-miRNA-mRNA network, we obtained basic information regarding the circRNAs, including their chromosomal locations from the circBase (http://www.circbase.org) [38]. We then used the cancer-specific circRNA database (CSCD) [39] to establish a circRNA-miRNA network associated with pancreatic tumorigenesis based on the intersection between the circRNA-miRNA pairs and the DEmiRNAs. We also downloaded all the available information of the circRNAs from the CSCD, including the number and the position of the microRNA response element (MRE), the RNA binding protein (RBP) and the open reading frame (ORF). Next, we obtained the miRNA-predicted mRNA pairs from the miRDB, miRTarBase and TargetScan databases, and made an intersection between the predicted mRNAs and the DEmRNAs to construct a miRNA-mRNA network related to pancreatic tumorigenesis. In the three databases, these predicted miRNA-mRNA pairs are validated experimentally by reporter assay, western blot, microarray and next-generation sequencing experiments. Only pairs existed in at least 2 out of 3 databases mentioned before were thought stable and would be included in the present study [40–42]. Finally, we constructed a visual display of the circRNA-miRNA-mRNA network using the Cytoscape software [43].

### Analysis of DEmRNAs

We performed GO enrichment analysis of the DEmRNAs to identify biological processes involved in pancreatic tumorigenesis using DAVID (version 6.8, Database for Annotation, Visualization and Integrated Discovery, https://david.ncifcrf.gov/) [44].

KEGG enrichment analysis was performed by the Gene Set Enrichment Analysis (GSEA) method using the "clusterprofiler" package [45]. Biological processes (BP) and KEGG pathways with a *P* value < 0.05 were considered statistically significant and visualized as a Sankey plot using the "ggalluvial" package [46].

### Identification and validation of the hub genes

We analyzed the relative importance of the DEmRNAs using the STRING database (https://string-db.org/) [47], which contains 9,643,763 proteins from 2,031 organisms and 1,380,838,440 interactions. We constructed the PPI network using the Cytoscape software. The genes were ranked using the cytoHubba plug-in in Cytoscape [48]. We identified the hub genes by integrating the results of the GO, KEGG and PPI network analyses. The expression of 4 circRNAs (*circ-HIBADH*, *circ-UBAP2*, *circ-TADA2A*, and *circ-CLEC17A*), 3 miRNAs (*has-miR-214*, *has-mir-324-3p*, and *has-miR-494*) and 5 hub genes (*CXCR4*,*HIF1A*, *ZEB1*, *SDC1* and *TWIST1*) were compared between the PAAD and the normal pancreatic tissues from the GEO dataset. The GEPIA (http://gepia.cancer-pku.cn/) [49] and THPA database (https://www.proteinatlas.org) [50] were used to validate the transcript (mRNA) and the protein levels of the hub genes. Differentially expressed genes were identified by applying the criteria of |log 2 FC| > 1 and P value < 0.05. Moreover, we compared the expression of the four circRNAs in men and women using the GSE79634 database that provides gender-based information for PAAD patients.

### Analysis of the hub genes

We plotted survival curve in KM plotter database (http://kmplot.com/analysis/) [51] to explore connections between overall survival (OS) and *5* hub genes (*CXCR4*, *HIF1A*, *ZEB1*, *SDC1* and *TWIST1)* as well as 3 miRNAs (*has-miR-214*, *has-mir-324-3p*, and *has-miR-494*) in the ceRNA network. Parameters as follows: split patients by “auto select best cutoff”, follow up threshold = 36 months, cancer type: pancreatic ductal adenocarcinoma. Significant results with *P* value < 0.05 were recoded.

The hub genes in the ceRNA network were then analyzed by the TIMER database (https://cistrome.shinyapps.io/ timer/) [52] to detect immune cell infiltration using various immune markers. The gene modules identified tumor-infiltrating immune cells such as B cells, CD4+ T cells, CD8+ T cells, neutrophils, macrophages, and DCs. The correlation modules identified the gene markers for CD8+ T cells, T cells (general), B cells, monocytes, TAMs, M1 and M2 macrophages, neutrophils, NK cells, DCs cells, Th1 cells, Th2 cells, Tfhs, Th17s cells, Tregs, and exhausted T cells that were previously reported [53]. The power of the correlation was determined as follows: “0.00–0.19” indicated very weak, “0.20–0.39” indicated weak, “0.40–0.59” indicated moderate, “0.60–0.79” indicated strong, and “0.80–1.0” indicated very strong.

## Supplementary Material

Supplementary Figures
