# Supplementary material for: CircUBAP2-mediated competing endogenous RNA network modulates tumorigenesis in pancreatic adenocarcinoma
Source: Aging (Albany NY). 2019 Oct 4;11(19):8484–501. doi: 10.18632/aging.102334 (PMC6814619; doi:10.18632/aging.102334)
Supplement: Supplementary Figures [file aging-11-102334-s001.pdf]

SUPPLEMENTARY FIGURES

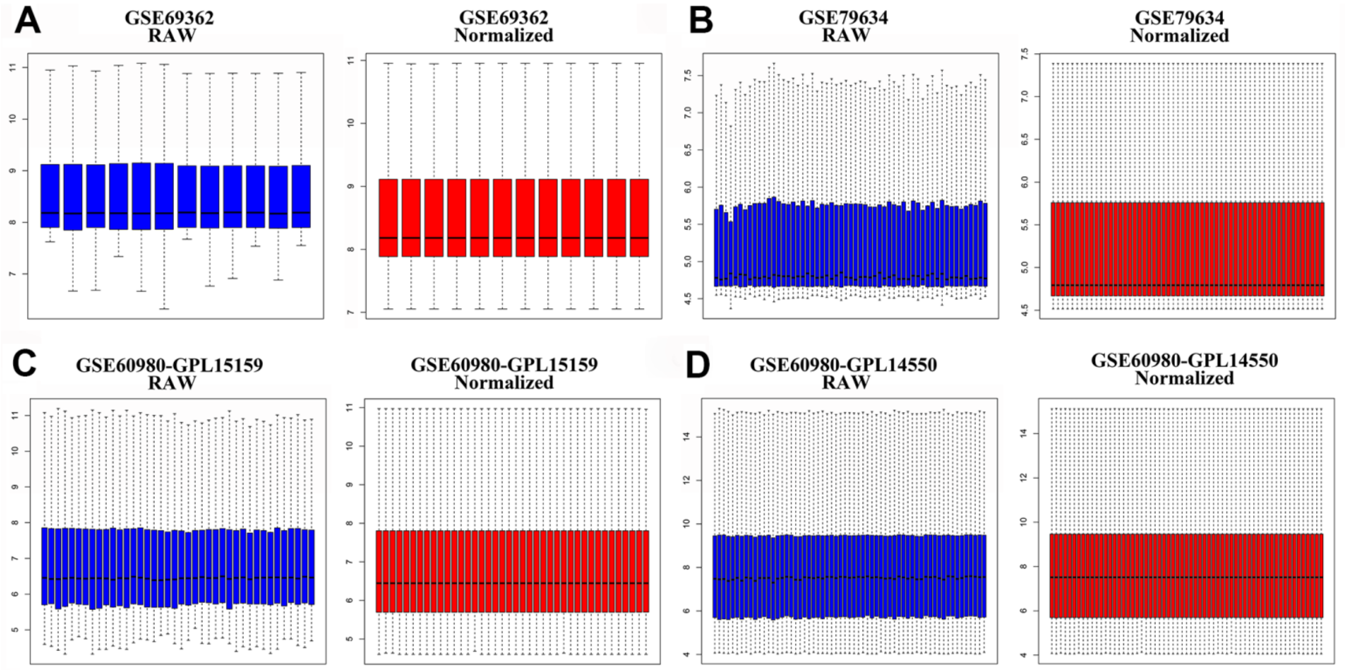

Supplementary Figure 1. Quality control of microarray data of circRNA, microRNA and mRNA. (**A**, **B**) The boxplots show the variations in the expression of circRNAs in the (**A**) GSE69362 and (**B**) GSE79634 datasets and their normalized data. (**C**, **D**) The boxplots show the variations in the expression of (**C**) microRNAs in the GSE60980 (GPL15159) dataset and (**D**) mRNAs in the GSE60980 (GPL14550) dataset and their normalized data.

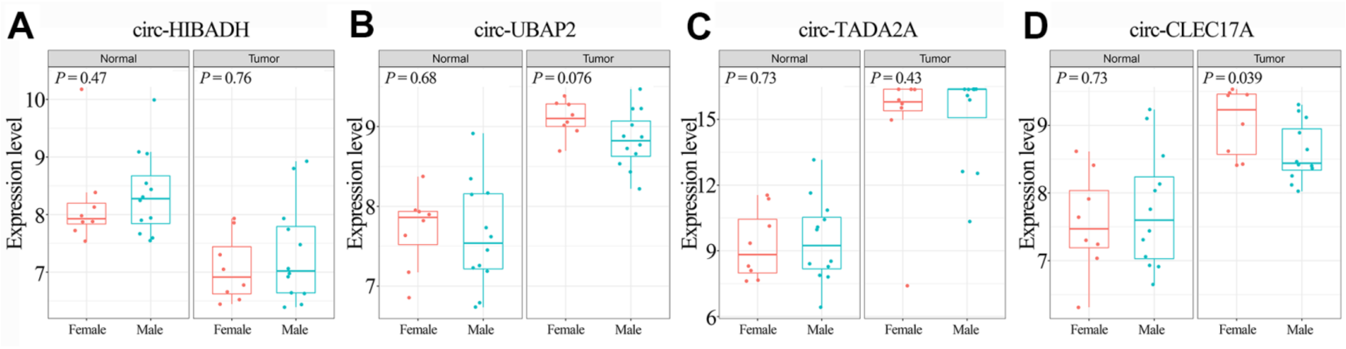

Supplementary Figure 2 Comparative analyses of the transcription levels of circRNAs between females and males in GSE69362. (**A**–**D**) Transcription levels of *circ-HIBADH*, *circ-UBAP2*, *circ-TADA2A*, and *circ-CLEC17A* in GSE69362 between females (red) and males (blue) in PAAD tumor tissues and normal pancreatic tissues.

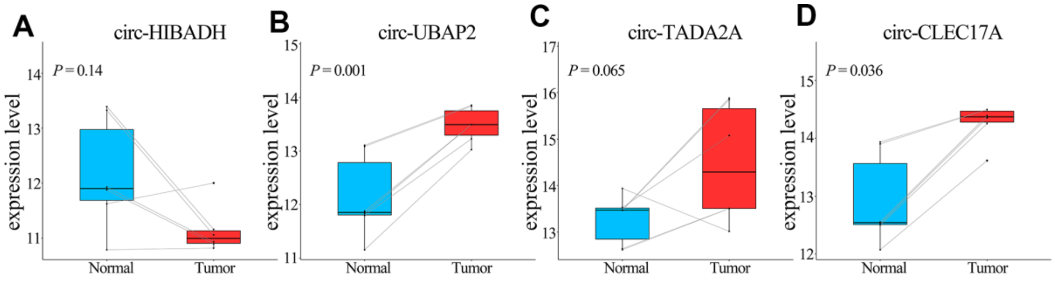

Supplementary Figure 3. Comparative analyses of the transcription levels of circRNAs between PAAD tumor tissues and normal pancreatic tissues in GSE69362. (**A**–**D**) Transcription levels of *circ-HIBADH*, *circ-UBAP2*, *circ-TADA2A* and *circ-CLEC17A* between PAAD tumor tissues (red) and normal pancreatic tissues (blue).

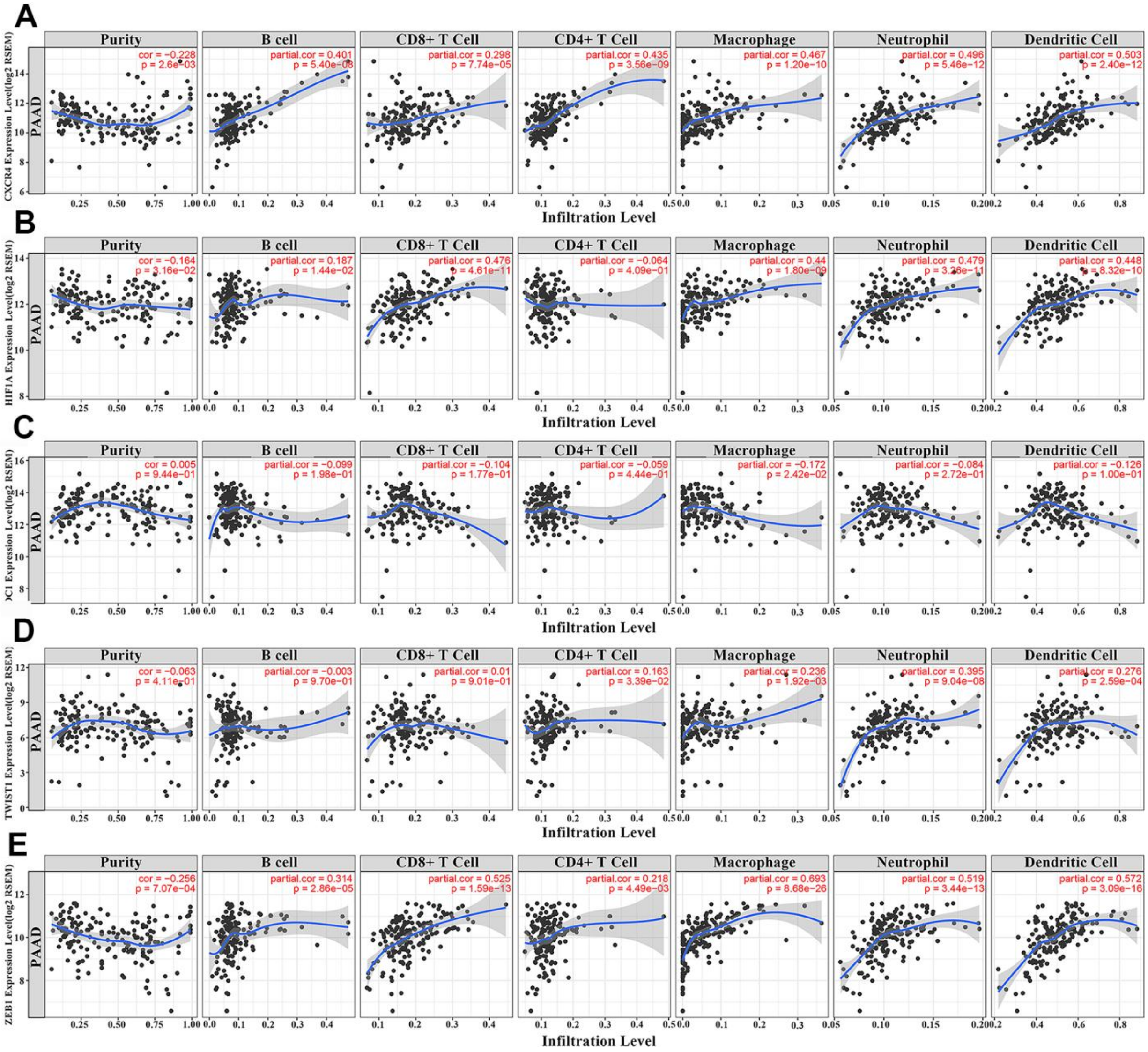

Supplementary Figure 4. The correlation between transcription levels of hub genes and the level of immune cell infiltration. The abscissa represents the abundance of immune cell infiltration, and the ordinate represents the transcription levels of the hub genes. The first column shows the correlation between transcription levels of hub genes and the purity of cancer cells. Columns from 2–7 show the purity-adjusted correlation between the transcription levels of hub genes and six different immune cells, including B cells, CD4 + T cells, CD8 + T cells, neutrophils, macrophages and dendritic cells. Hub genes: (**A**) *CXCR4*, (**B**) *HIF1A*, (**C**) *SDC1*, (**D**) *TWIST1*, (**E**) *ZEB1*.
